# Molecular identification of two newly identified human pathogens causing leishmaniasis using PCR-based methods on the 3′ untranslated region of the heat shock protein 70 (type I) gene

**DOI:** 10.1371/journal.pntd.0009982

**Published:** 2021-11-30

**Authors:** Narissara Jariyapan, Michelle D. Bates, Paul A. Bates

**Affiliations:** 1 Department of Parasitology, Faculty of Medicine, Chulalongkorn University, Bangkok, Thailand; 2 Division of Biomedical and Life Sciences, Faculty of Health and Medicine, Lancaster University, Lancaster, United Kingdom; CSIR-Indian Institute of Chemical Biology, INDIA

## Abstract

PCR-based methods to amplify the 3′ untranslated region (3′-UTR) of the heat shock protein 70 (type I) gene (*HSP70-I*) have previously been used for typing of *Leishmania* but not with *Leishmania* (*Mundinia*) *martiniquensis* and *L*. (*Mundinia*) *orientalis*, newly identified human pathogens. Here, the 3′-UTRs of *HSP70-I* of *L*. *martiniquensis*, *L*. *orientalis*, and 10 other species were sequenced and analyzed. PCR-Restriction Fragment Length Polymorphism (RFLP) analysis targeting the 3′-UTR of *HSP70-I* was developed. Also, the detection limit of *HSP70-I*-3′-UTR PCR methods was compared with two other commonly used targets: the 18S small subunit ribosomal RNA (SSU-rRNA) gene and the internal transcribed spacer 1 region of the rRNA (ITS1-rRNA) gene. Results showed that *HSP70-I*-3′-UTR PCR methods could be used to identify and differentiate between *L*. *martiniquensis* (480–2 bp) and *L*. *orientalis* (674 bp) and distinguished them from parasites of the subgenus *Viannia* and of the subgenus *Leishmania*. PCR-RFLP patterns of the 3′-UTR of *HSP70-I* fragments digested with *Bsu*RI restriction enzyme successfully differentiated *L*. *martiniquensis*, *L*. *orientalis*, *L*. *braziliensis*, *L*. *guyanensis* = *L*. *panamensis*, *L*. *mexicana* = *L*. *aethiopica = L*. *tropica*, *L*. *amazonensis*, *L*. *major*, and *L*. *donovani* = *L*. *infantum*. For the detection limit, the *HSP70-I*-3′-UTR PCR method could detect the DNA of *L*. *martiniquensis* and *L*. *orientalis* at the same concentration, 1 pg/μL, at a similar level to the SSU-rRNA PCR. The PCR that amplified ITS1-rRNA was more sensitive (0.01 pg/μL) than that of the *HSP70-I*-3′-UTR PCR. However, the sizes of both SSU-rRNA and ITS1-rRNA PCR amplicons could not differentiate between *L*. *martiniquensis* and *L*. *orientalis*. This is the first report of using *HSP70-I*-3′-UTR PCR based methods to identify the parasites causing leishmaniasis in Thailand. Also, the *Bsu*RI-PCR-RFLP method can be used for differentiating some species within other subgenera.

## Author summary

*L*. *martiniquensis* and *L*. *orientalis*, newly identified human pathogens, cause visceral leishmaniasis and cutaneous leishmaniasis in HIV-negative patients, respectively. However, both parasite species cause disseminated cutaneous leishmaniasis accompanying visceral leishmaniasis in HIV-positive patients. Species typing in leishmaniasis is important in diagnostics, epidemiology, and clinical studies. We show here that the 3′-UTR of *HSP70-I* region is a suitable target for PCR-based identification and discrimination between *L*. *martiniquensis* and *L*. *orientalis*. The technique is simple to perform and can be implemented in all settings where PCR is available. In species with similar PCR product size, the *Bsu*RI-PCR-RFLP patterns of the 3′-UTR of *HSP70-I* fragments can be used for differentiating some species within other subgenera. However, where identification of species is essential or there is a travel history outside Thailand, sequencing of the *HSP70-I*-3′-UTR product or a similar discriminating target sequence is recommended. The PCR-based methods used in this study can also be applicable to the identification of *Leishmania* species obtained from vectors and reservoirs.

## Introduction

Leishmaniasis is a newly emerging disease in Thailand. To date two species of *Leishmania* have been reported from Thailand: *L*. (*Mundinia*) *martiniquensis* [1] and *L*. (*Mundinia*) *orientalis* [2]. There has been one report of *L*. *infantum* but this may have been an imported case [3]. The clinical signs of *L*. *martiniquensis* infection include both cutaneous and visceral leishmaniasis in both HIV-infected and immunocompetent patients [1,4]. One case of *L*. *orientalis* presented as simple cutaneous leishmaniasis in an immunocompetent patient, and two others as disseminated cutaneous/visceral leishmaniasis in HIV-infected patients [2].

Identification of the species responsible for a *Leishmania* infection is important for clinical management, treatment, and epidemiology. Although there can be substantial overlap in symptoms, each species will cause its own spectrum of disease manifestations that require their own supportive measures and have different prognoses. For example, *L*. *major* and *L*. *tropica* both cause cutaneous leishmaniasis in the majority of patients and can occur sympatrically in many regions of North Africa and the Middle East but have different epidemiology [5]. *L*. *major* is exclusively zoonotic in origin and skin lesions infection will typically resolve, whereas *L*. *tropica* tends to occur in epidemics driven by anthroponotic transmission, with lesions that are more persistent or reactivate (leishmaniasis recidivans). Another example is cutaneous leishmaniasis in Central and South America where infections caused by *L*. *amazonensis* and *L*. *braziliensis* can occur in the same areas, but the consequences of infection are potentially very different. *L*. *amazonensis* mostly causes simple cutaneous leishmaniasis but can also progress to disseminated cutaneous leishmaniasis with multiple lesions, whereas *L*. *braziliensis* will sometimes progress to destructive mucocutaneous disease [6,7].

Although multilocus enzyme electrophoresis (MLEE) of cultured parasites was the best approach for *Leishmania* species identification for many years [8], it is a time-consuming procedure and requires mass parasite culturing. However, more critically, it has now become apparent that strains with apparently the same enzyme phenotype can have distinct amino acid sequences, and putative heterozygous phenotypes are also difficult to interpret [9]. Therefore, MLEE has become superseded by molecular approaches such as PCR and DNA sequencing, which have the potential to be more sensitive, rapid and easier to perform. Results are available within days versus weeks or months, and in the case of sequencing provide a permanent record available to all researchers. So far, several target genes have been used in PCR and sequencing approaches for *Leishmania* species identification, such as the SSU-rRNA gene [10], the RNA polymerase II gene [11], the DNA polymerase α gene [12], gp63 genes [13], the cytochrome oxidase II gene [14,15], spliced leader mini-exon genes [16], the glucose-6-phosphate dehydrogenase (G6PD) gene [17], the heat shock protein 70 (HSP70) gene [18], 18S ribosomal RNA [19], the cysteine protease b (cpb) gene [20], the N-acetylglucosamine-1-phosphate transferase gene [21], the internal transcribed spacer 1 (ITS1) region of the SSU-rRNA gene [22,23], the cytochrome b (cyt b) gene [24], 7SL RNA genes [25], and triose-phosphate isomerase (tim) genes [26].

For the species found in Thailand, so far only one PCR-based method, using a pair of primers for minicircle kinetoplast DNA [27], has been used to discriminate *L*. *martiniquensis* from ‘*L*. *siamensis*’ (MHOM/TH/2010/TR) (syn. *L*. *orientalis*), but the size of the PCR products could not differentiate these parasites from other subgenera [28]. In 2012, Requena and colleagues analyzed the 3′untranslated region (3′UTR) of *Leishmania* HSP70-type I (*HSP70-I*) genes from 24 strains representing eleven *Leishmania* species following PCR amplification [29]. It was observed that there was sufficient sequence conservation in the primer targets to enable amplification of DNA from species of both the subgenus *Leishmania* and *Viannia*. This particular region of the *HSP70-I* gene appears to have the potential to discriminate between *Leishmania* subgenera by direct visualization of different sizes of PCR amplification products [29]. The main objective of this study was to use the 3′UTR region of the *HSP70-I* gene to identify and diagnose *L*. *martiniquensis* and *L*. *orientalis*, thereby expanding the species range to the subgenus *Mundinia* [30] and including the species found in Thailand. In addition to direct PCR, *HSP70-I*-3′-UTR restriction fragment length polymorphism (*HSP70-I*-3′-UTR PCR-RFLP) was also performed to investigate the potential of this method to identify *Leishmania* species. In addition, the detection limit of the *HSP70-I*-3′-UTR PCR was compared with that of two other widely used targets: the SSU-rRNA gene and the ITS1 of the rRNA gene.

## Materials and methods

### Ethics statement

The study was approved by the ethics committee of the Faculty of Medicine, Chulalongkorn University (IRB number: 051/64). No patient information is presented in this study.

### Parasite isolates and culture

The following species isolates were grown as promastigotes *in vitro* as previously described [1]: *L*. *aethiopica* (LV546); *L*. *amazonensis* (M2269); *L*. *braziliensis* (U1096); *L*. *donovani* (LV9); *L*. *guyanensis* (M4147); *L*. *infantum* (JPC); *L*. *major* (FV1); *L*. *martiniquensis* (LSCM1, LSCM2, LSCM3, LSCM5, LEM2494); *L*. *mexicana* (M379); *L*. *orientalis* (LSCM4); *L*. *panamensis* (LS94); *L*. *tropica* (LV357).

### Isolation of DNA

*Leishmania* species and human blood DNAs were extracted using a QIAamp DNA Mini Kit (Qiagen, Hilden, Germany) according to the manufacturer’s instructions. DNA concentration was measured using a Nano Drop spectrophotometer (ND-1000 model, Fisher Scientific, Loughborough, UK).

### PCR and sequencing

The *HSP70-I*-3′-UTR amplification using primers 70-IR-D (5′- CCAAGGTCGAGGAGGTCGACTA -3′) and 70-IR-M (5′- ACGGGTAGGGGGAGGAAAGA -3′) [29] was performed with proof-reading DNA polymerase (Qiagen HotStar HiFidelity Polymerase, Qiagen, USA). PCR was performed in a final volume of 50 μL, containing 50 pmol of each primer, 10 μL of 5X Qiagen PCR buffer, 1 μL of DNA polymerase and 1 μL (40 pg) of each DNA template, using the following amplification cycle: 95°C for 2 min followed by 30 cycles of 95°C for 30 sec, 62.5°C for 30 sec, and 72°C for 1 min and 20 sec, and a final extension at 72°C for 5 min [29]. Distilled water was used as the negative control. Expected amplicons of PCR products were separated on 1.5% agarose gels, stained with GelRed (Thermo Fisher Scientific, Loughborough, UK), and visualized using a GelDoc imaging system (Ultra-Violet Products Ltd., Cambridge, UK). PCR products were purified using a PCR purification kit (Thermo Fisher Scientific, Loughborough, UK) and directly sequenced using commercial services (Source Bioscience Sequencing, Cambridge, UK), and checked for quality using Chromas Lite 2.1.1 (http://technelysium.com.au/). Sequence alignments were performed using Clustal Omega (http://www.ebi.ac.uk/tools/msa/clustalo/).

### *In silico* restriction analysis

*In silico* analyses of predicted *Bsu*RI restriction fragments of the 3′-UTR sequences of *HSP70-I* genes were performed using RestrictionMapper version 3 (www.restrictionmapper.org/) and theoretical fragment sizes were determined manually. Microsatellite distribution in the 3′-UTR of *HSP70-I* genes was analyzed manually.

### PCR-RFLP analysis

PCR amplifications targeting the 3′-UTR sequences of *HSP70-I* genes were performed as described above. The PCR products were digested with a restriction enzyme, *Bsu*RI, (Thermo Fisher Scientific Baltics UAB, Vilnius, Lithuania) according to the manufacturer’s instruction. *Bsu*RI is an isoschizomer of *Hae*III, which has been previously used to analyze *Leishmania* by PCR-RFLP [16,18], both cutting the target sequence GGCC. Patterns of digestion products were analyzed by 3% agarose gel electrophoresis. The GeneRuler 100 bp DNA ladder (Thermo Fisher Scientific Baltics UAB, Vilnius, Lithuania) was used as a DNA size marker. Gels were stained with GelRed (Thermo Fisher Scientific, Loughborough, UK), and visualized using a GelDoc imaging system (Ultra-Violet Products Ltd., Cambridge, UK).

### Detection limits of the PCR method

Ten-fold serial dilutions of the extracted *Leishmania* DNAs were prepared to generate standard concentrations at 100,000; 10,000; 1,000; 100; 10; 1; 0.1; 0.01; 0.001; and 0.0001 pg/μL. To mimic a real situation of the detection of DNA of parasites extracted from blood or tissue samples, human DNA extracted from blood at a concentration of 18 ng/μL was used as a background DNA and diluent in the 10-fold dilution series of the *Leishmania* DNA. One microliter of each concentration was used as a DNA template for PCR amplification in a final volume of 25 μL. Each PCR reaction contained 100,000 to 0.0001 pg of *Leishmania* DNA and 19.8 ng of human DNA. PCR was performed on the 3′-UTR sequences of *HSP70-I* genes as described above and these results compared with two other commonly used identification targets as follows. *Leishmania*-specific primers R221 (5′-GGTTCCTTTCCTTGATTTAGC-3′) and R332, (5′-GGCCGGTAAAGGCCGAATAG-3′) were used to amplify a region of the 18S rRNA gene, generating a product of 603 bp [31,32]. Primers LeR (5^′^-CCAAGTCATCCATCGCGACACG-3^′)^ and LeF (5^′^-TCCGCCCGAAAGTTCACCGATA-3^′^) were used to amplify an internal transcribed spacer 1 region (ITS1) of the rRNA gene, generating a product of 379 bp [22]. To confirm the presence of a standard concentration of background human DNA universal primers UNFOR403 (5’-TGAGGACAAATATCATTCTGAGG-3’) and UNREV1025 (5’-GGTTGTCCTCCAATTCATGTTA-3’) were used to amplify the human DNA [33]. PCR products were run on 1.5% agarose gels, stained with GelRed (Thermo Fisher Scientific, Loughborough, UK), and visualized using a GelDoc imaging system (Ultra-Violet Products Ltd., Cambridge, UK).

### Evaluation of the assay on DNA from clinical samples

To test this PCR assay on DNA extracted directly from clinical samples, six DNA samples extracted from saliva, blood, and skin of *L*. *martiniquensis* cases [34,35] were used.

## Results

### PCR amplification and sequencing of 3′-UTR regions of *Leishmania HSP70-I* genes

The 70-IR-D and 70-IR-M primers were used to amplify the *HSP70-I*-3′-UTR DNA from five previously characterized isolates of *Leishmania* from northern Thailand. Four of them were *L*. *martiniquensis* (LSCM1, LSCM2, LSCM3, LSCM5) and all of these produced a band of ~480 bp on agarose gel electrophoresis, similar to the reference isolate LEM2494 (S1 Fig). Another isolate was *L*. *orientalis* (LSCM4; reference isolate). It produced a band of ~670 bp. The PCR products of the five Thai isolates were sequenced. The sequences of the four isolates of *L*. *martiniquensis* were aligned and showed a high degree of similarity with each other and the reference isolate LEM2494 (99.5–100% identity) (S2 Fig).

**Fig 1:**
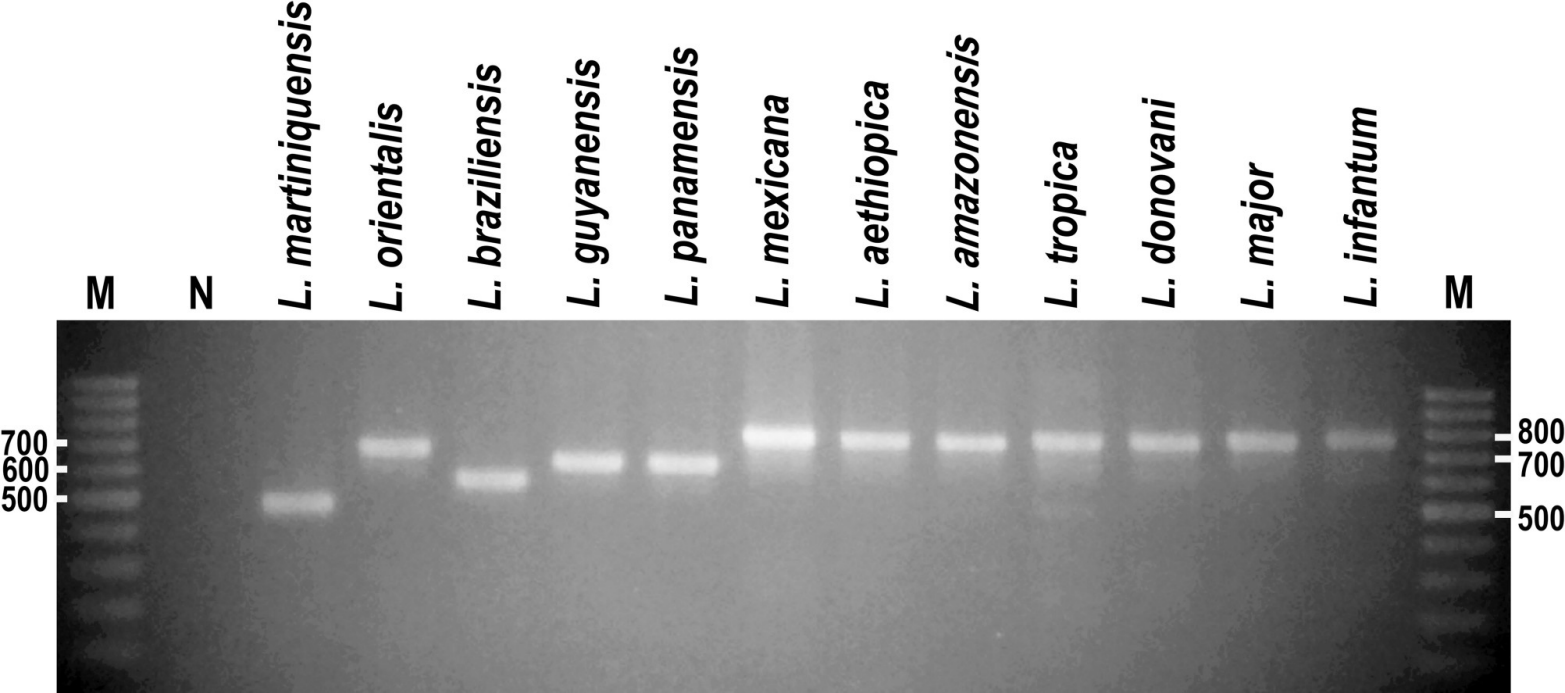
Agarose gel electrophoresis of *HSP70-I*-3′-UTR PCR products of 12 *Leishmania* species. M = Molecular markers and N = Negative control.

In addition to these, *HSP70-I*-3′-UTR PCR products were generated from 10 other species of *Leishmania*: *L*. *aethiopica*, *L*. *amazonensis*, *L*. *braziliensis*, *L*. *donovani*, *L*. *guyanensis*, *L*. *infantum*, *L*. *major*, *L*. *mexicana*, *L*. *panamensis*, and *L*. *tropica*. These PCR products were variable in size, but all could be clearly discriminated from the small ~480 bp product of *L*. *martiniquensis* using gel electrophoresis (Fig 1). The ~670 bp product of *L*. *orientalis* was also a different size to those of the other species but would be quite difficult to discriminate by electrophoresis alone. Parasites of the subgenus *Viannia* (*L*. *braziliensis*, *L*. *guyanensis*, and *L*. *panamensis*) produced products of ~550–630 bp, whereas the remaining species all in subgenus *Leishmania* produced larger products of ~750–780 bp (Fig 1). To confirm these results, these *HSP70-I*-3′-UTR PCR products were all sequenced, and the exact sizes of these sequences are shown in Table 1, together with their GenBank accession numbers.

**Table 1:** *In silico* prediction of the *HSP70-I*-3′-UTR*-Bsu*RI restriction fragments and fragment sizes in PCR-RFLP.

*Leishmania* species	Isolates	Accession number	PCR product size (bp)	*Bsu*RI restriction fragments	Fragment sizes (bp) by *in silico* prediction	Fragment sizes (bp) on 3% agarose gel
*L*. *martiniquensis*	MHOM/MQ/1992/MAR1;LEM2494	MK607435	482	3	343, 131, 8	ND
	MHOM/TH/2012/LSCM1	MK607436	480	3	341, 131, 8	341, 131
	MHOM/TH/2013/LSCM2	MK60737	482	3	343, 131, 8	ND
	MHOM/TH/2013/LSCM3	MK60738	480	3	341, 131, 8	ND
	MHOM/TH/2015/LSCM5	MK60739	480	3	341, 131, 8	ND
*L*. *orientalis*	MHOM/TH/2014/LSCM4	MK607444	674	4	300, 286, 82, 6	300–286^a^, 82
*L*. *braziliensis*	MHOM/GT/2001/U1096	MK607440	562	4	330, 104, 81, 47	330, 104–81^a^
*L*. *guyanensis*	MHOM/BR/1975/M4147	MK607441	627	3	442, 104, 81	>500, 442, 104–81^a^
*L*. *panamensis*	MHOM/PA/1971/LS94	MK607442	629	3	444, 104, 81	>500, 442, 104–81^a^
*L*. *mexicana*	MNYC/BZ/1962/M379	MK607446	738	3	325, 237, 176	325, 237, 176
*L*. *aethiopica*	MHOM/ET/1972/L100;LV546	HE575327	742	3	321, 243, 178	321, 243, 178
*L*. *amazonensis*	MHOM/BR/1997/M2269	MK607447	740	3	413, 216, 111	413, 216, 111
*L*. *tropica*	MHOM/IR/1960/LV357	MK607448	746	3	321, 243, 182	321, 243, 182
*L*. *donovani*	MHOM/ET/1967/HU3;LV9	HE575332	753	4	437, 165, 124, 27	437, 165, 124
*L*. *major*	MHOM/IL/1980/FV1	MK607449	759	4	323, 188, 178, 70	323, 188–178^a^, 70
*L*. *infantum*	MCAN/ES/1998/JPCM5	MK607450	773	4	436, 182, 128, 27	436, 182, 128

ND = not done

^a^Merged band from 2 DNA fragments that were different in size approximately 20 bp or less.

### The *HSP70-I*-3′-UTR PCR assay on DNA extracted from clinical samples

**Fig 2:**
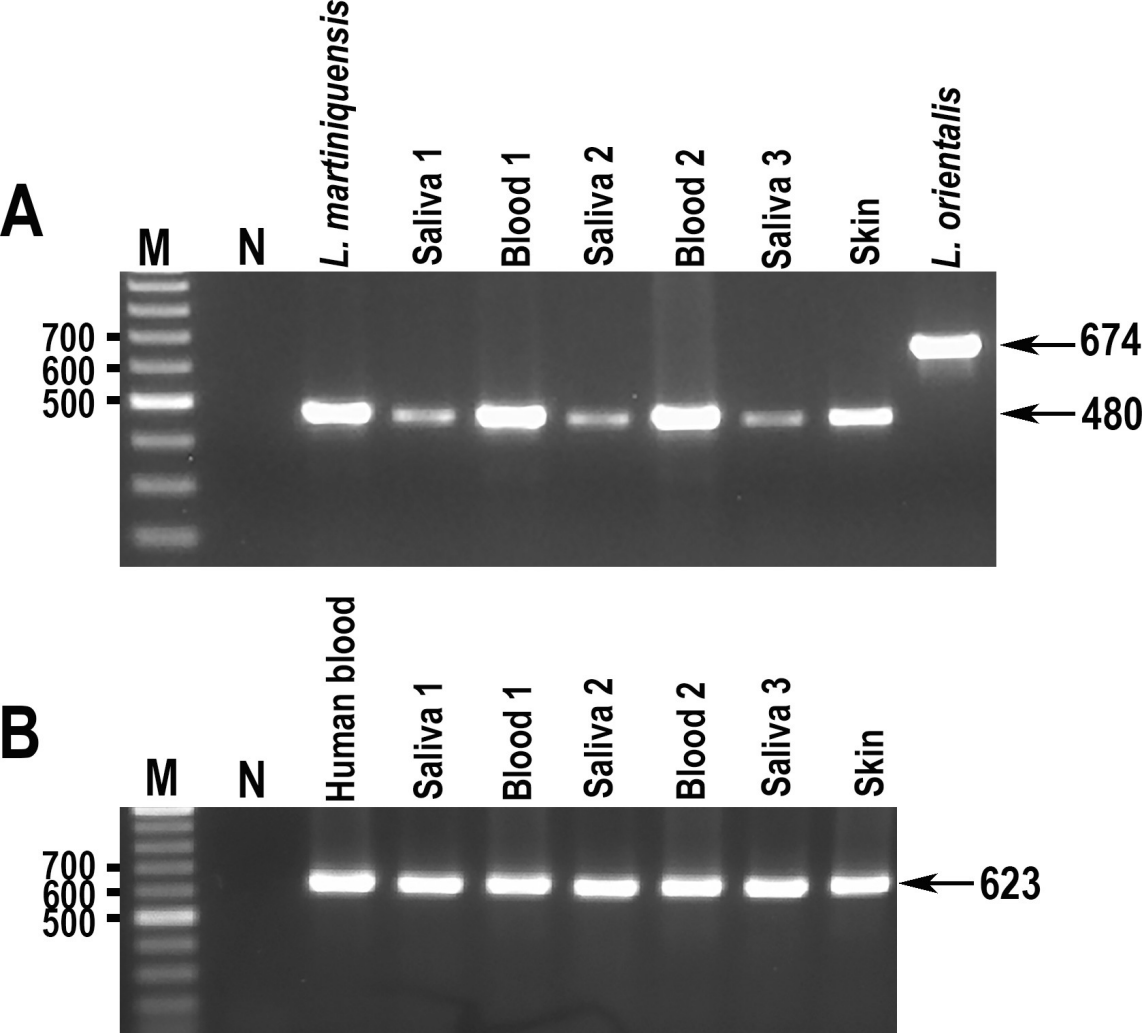
Agarose gel electrophoresis of *HSP70-I*-3′-UTR PCR products of DNA extracted from clinical samples. A. *HSP70-I*-3′-UTR PCR products B. PCR products amplified using UNFOR403 and UNREV1025 primers. M = Molecular markers and N = Negative control.

The *HSP70-I-*3′-UTR PCR was applied to six DNA samples extracted from clinical specimens: three from saliva; two from blood; and one from skin of *L*. *martiniquensis* cases [34,35]. PCR products were successfully generated in each case and, as expected, the size of these products from saliva, blood, and skin specimens completely matched with the size of the *L*. *martiniquensis* PCR product (Fig 2).

### The 3′-UTRs of *HSP70-I* genes contain various microsatellites

**Fig 3:**
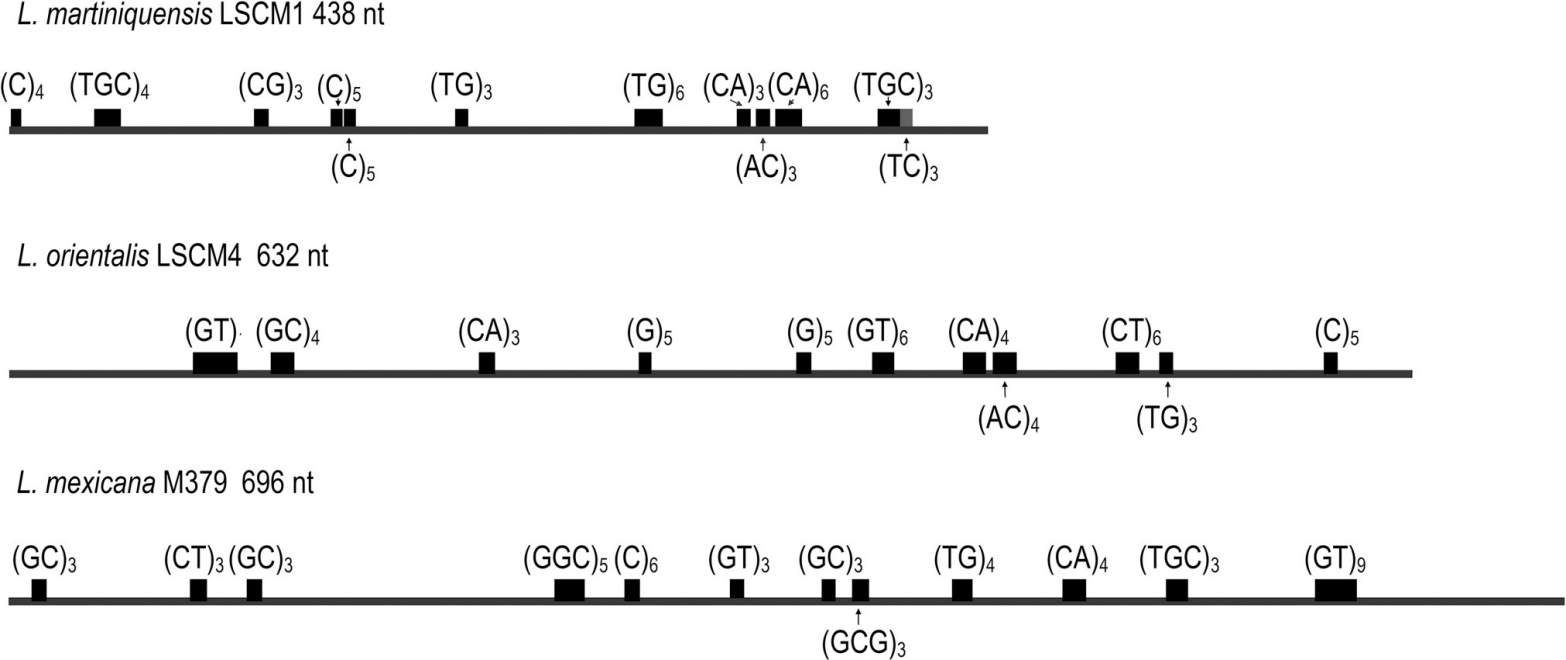
Microsatellite distribution in 3’-UTR of *HSP70-I* genes of *L*. *martiniquensis*, *L*. *orientalis*, and *L*. *mexicana*. The repeated motifs correspond to those found in the sense strand. Note that the drawing scale is not proportional for the different *Leishmania* species.

One source of size variation in untranslated regions is the occurrence of microsatellites. Since the 3′-UTRs of *HSP70-I* genes in *L*. *martiniquensis*, *L*. *orientalis*, and *L*. *mexicana* have not been analyzed for microsatellites, a search for structural motifs along the sequenced fragments of the three species was performed. The results show that the 3′-UTR of *HSP70-I* fragments contained many microsatellites. The most common microsatellite was CA repeats (as TG or GT on the complementary strand). However, TGC-microsatellites were not found in *L*. *orientalis* (Fig 3).

### *In silico* prediction of *Bsu*RI restriction fragments of 3′-UTR sequences of *HSP70-I* genes and PCR-RFLP analysis

To explore the potential for enhanced discrimination between species by PCR-RFLP, *in silico* analyses of *Bsu*RI restriction fragments of the 3′-UTR sequences of *HSP70-I* genes of *L*. *martiniquensis*, *L*. *orientalis* and the 10 other species were performed. This revealed a variety of different predicted patterns and fragment sizes (Table 1). We also analyzed *L*. *mexicana* for the first time, and the *in silico* analysis showed different fragment sizes from most other species, but in this respect was similar to *L*. *aethiopica* and *L*. *tropica*.

**Fig 4:**
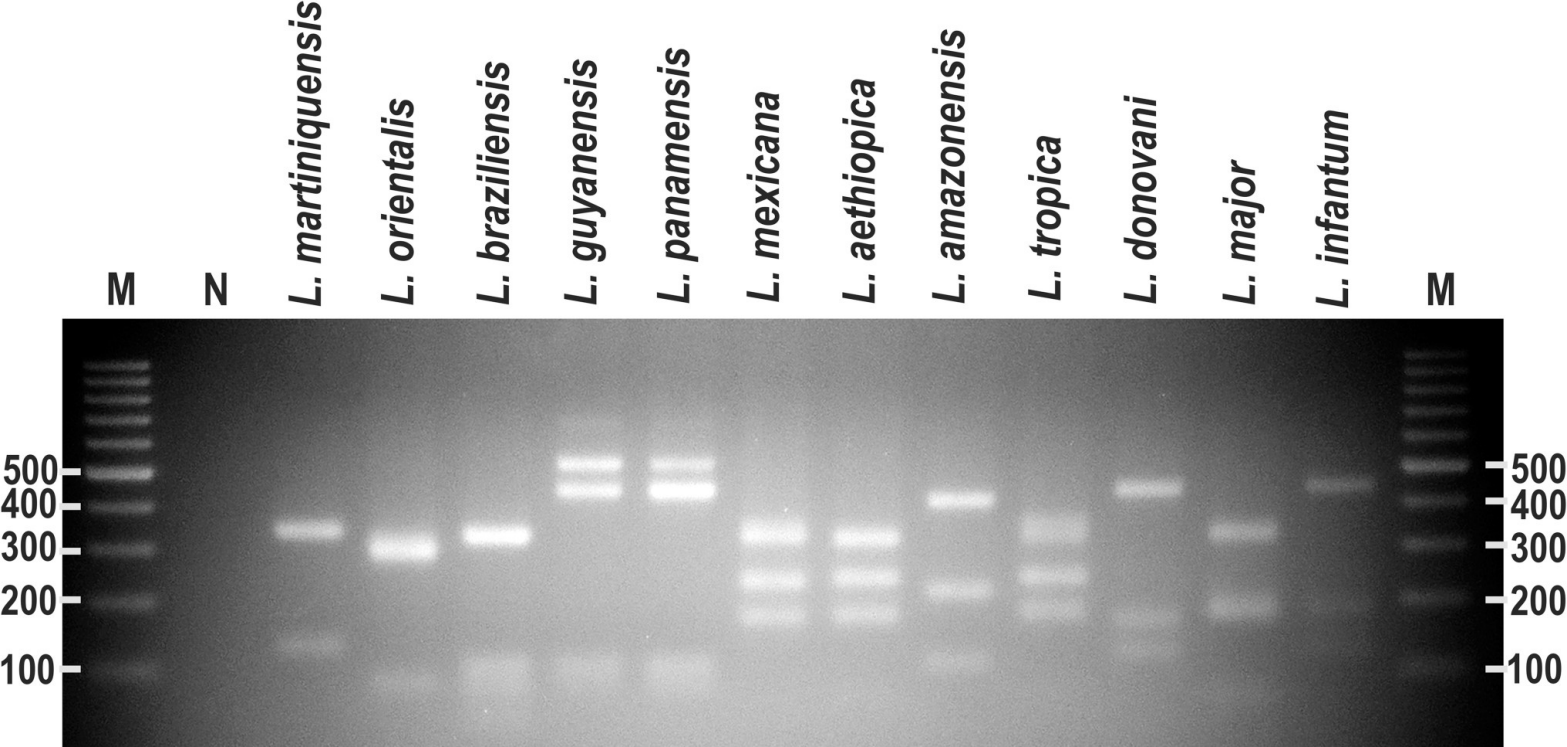
PCR-RFLP analyses of *HSP70-I*-3′-UTR fragments of 12 *Leishmania* species. PCR amplification was performed with the *HSP70-I*-3′-UTR-specific primers, and PCR products were digested with *Bsu*RI. M = Molecular markers and N = Negative control.

To test the *in silico* prediction, PCR-RFLP analysis targeting the 3′-UTR of *HSP70-I* following by digesting with *Bsu*RI restriction enzyme was performed for the 12 *Leishmania* species. Different PCR-RFLP patterns of digested 3′-UTR of *HSP70-I* amplicons with *Bsu*RI were obtained for *L*. *martiniquensis*, *L*. *orientalis*, *L*. *braziliensis*, *L*. *guyanensis* = *L*. *panamensis*, *L*. *mexicana* = *L*. *aethiopica* = *L*. *tropica*, *L*. *amazonensis*, *L*. *donovani* = *L*. *infantum*, and *L*. *major* (Fig 4 and Table 1). The PCR-RFLP patterns of *L*. *guyanensis* (M4147) and *L*. *panamensis* (LS94) were similar and one extra band (>500 bp) was observed differentially from the *in silico* predicted patterns. Results also confirm that *L*. *mexicana*, *L*. *aethiopica* and *L*. *tropica* had similar PCR-RFLP patterns (Fig 4 and Table 1). Likewise, *L*. *donovani* and *L*. *infantum* had similar banding patterns. Some of the fragments that were similar in size were below the resolution limit and could not be discriminated by gel electrophoresis, appearing as merged bands. These are indicated in Table 1.

Of these results perhaps the most surprising is the similarity in banding pattern of *L*. *mexicana* to *L*. *aethiopica*/*L*. *tropica* and difference to *L*. *amazonensis*. To confirm this was a genuine result and not due to a mix up of cultures further sequence analysis was performed on three *L*. *amazonensis* and two *L*. *mexicana* isolates (S3 Fig). This analysis confirmed the close sequence similarity between all five of these isolates and the reason for the banding patterns observed. The two *L*. *mexicana* isolate sequences were identical to each other and the three *L*. *amazonensis* isolates showed 95–99% identity. All five isolates had a conserved *Bsu*RI site, but due to single nucleotide polymorphisms and insertions, an additional site found in all three *L*. *amazonensis* isolates was absent in both *L*. *mexicana* isolates, and conversely an additional site in both *L*. *mexicana* isolates was absent from all three *L*. *amazonensis* isolates. These account for the difference in banding pattern between *L*. *amazonensis* and *L*. *mexicana*. The analysis shows that the similar sized bands observed in *L*. *mexicana* and *L*. *aethiopica*/*L*. *tropica* is a result of co-incidence rather than overall sequence similarity.

### Detection limits of three PCR methods to differentiate between *L*. *martiniquensis* and *L*. *orientalis*

**Fig 5:**
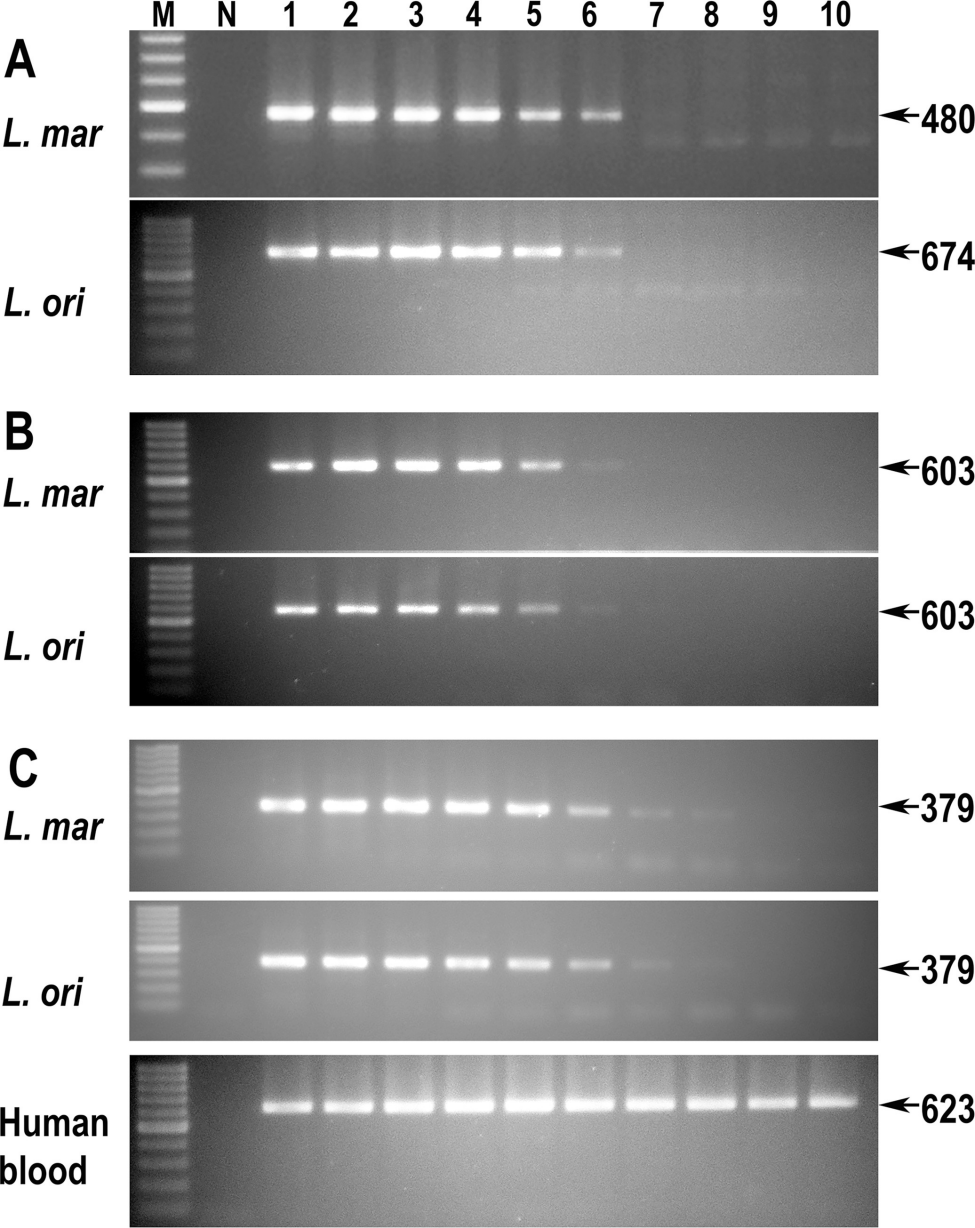
Agarose gel electrophoresis of PCR products of *L*. *martiniquensis* and *L*. *orientalis*. A. *HSP70-I*-3′-UTR PCR products B. SSU-rRNA PCR products C. ITS1-rRNA PCR products. Human blood DNA at 19.8 ng per reaction was used as a background control. N = negative control and lanes 1–10 indicate amounts of 100,000; 10,000; 1,000; 100; 10; 1; 0.1; 0.01; 0.001; and 0.0001 pg of *Leishmania* DNA in each reaction, respectively.

Finally, for *L*. *martiniquensis* and *L*. *orientalis* we investigated the detection limit of the *HSP70-I*-3′-UTR PCR method and compared this target with two other widely used sequences, SSU-rRNA and ITS1-rRNA. The *HSP70-I*-3′-UTR method could detect *L*. *martiniquensis* and *L*. *orientalis* at the same DNA concentration which was 1 pg/μL (Fig 5A). Compared to this, the PCR amplified SSU-rRNA regions showed similar detection limits in both *Leishmania* species (Fig 5B). The ITS1-rRNA PCR method could detect the DNA of both *Leishmania* species at a lower concentration of 0.01 pg/μL (Fig 5C). However, note that the sizes of both SSU-rRNA and ITS1-rRNA PCR amplicons could not differentiate between *L*. *martiniquensis* and *L*. *orientalis*.

## Discussion

Species identification in leishmaniasis is important in diagnostics, epidemiology, and clinical studies. To that end, DNA sequencing is an effective method used to identify *Leishmania* species. However, in some remote areas of Thailand and other low income or developing countries in Southeast and East Asia or elsewhere, it is not a cost-effective method and is not feasible for routine diagnosis, and sending samples elsewhere for sequencing has other problems, especially in transportation. Simple PCR methods would be preferred for diagnosis and identification of the pathogenic parasites and also medical follow-up. The current study demonstrated that PCR of the *HSP70-I*-3′-UTR region was able to generate products that could readily distinguish between *L*. *martiniquensis* (480–2 bp) and *L*. *orientalis* (674 bp), the two major causative agents of leishmaniasis in Thailand, on the basis of their size alone, providing this is accurately measured. To confirm the application of the *HSP70-I-*3′-UTR PCR method to clinical samples, DNA extracted from saliva, blood, and skin, were used. The positive results indicate that the PCR method can be applied to DNA extracted from clinical samples in general. Although we did not test with DNA extracts from clinical samples of *L*. *orientalis* because no materials were available, the sensitivity testing shows that the PCR was able to identify DNA down to 1 pg/uL, which in practice is certainly useful for diagnosis. Such information is clinically useful, as from the cases known to date *L*. *martiniquensis* appears capable of causing more serious disease than *L*. *orientalis*. However, information on possible intra-specific diversity of *L*. *orientalis HSP70-I*-3′-UTR is limited as only one *L*. *orientalis* isolate [2] was cultured and available for this study. If more *L*. *orientalis* isolates are obtained in the future, intra-specific diversity of the region can be investigated. Therefore, PCR alone can be used to confirm a suspected autochthonous case of leishmaniasis in Thailand and could provisionally identify the species.

Analysis of the HSP70-I sequences generated in this and previous studies has shown that the 3′-UTR region in *Leishmania* species of the subgenus *Leishmania* was clearly the longest. For the subgenus *Mundinia*, the UTR regions of all species except *L*. *martiniquensis* were longer than species of the subgenus *Viannia*. The UTR region of *L*. *martiniquensis* was the shortest. The biological significance of this size variation, if any, is unclear, but it might influence adaptation of the parasites in different environments/hosts as the 3′-UTR contains various sequences of regulatory regions that are involved in post-transcription of *HSP70* expression. The HSP70 protein assists the *Leishmania* parasites in survival within host macrophages and adaptation to the new environment following infection and tolerance against different stresses during their life cycle [36]. Further study on the regulation of the *HSP70*-*I* expression in the new species in the subgenus *Mundinia* should be performed, especially *L*. *martiniquensis*, as it can be a pathogen in various hosts in several regions of the world [1,37–39].

Microsatellites are repeated motifs of about one to six non-coding nucleotides ubiquitously distributed in all eukaryotic genomes [40]. Microsatellites of the *HSP70-I*-3′-UTR fragments of *L*. *martiniquensis*, *L*. *orientalis*, and *L*. *mexicana* were reported here for the first time. The most common microsatellite found, CA repeats, was observed in the 3′-UTR of all studied species. This is consistent with the reports in *L*. *infantum*, *L*. *major* and *L*. *braziliensis* genomes [29,41]. However, another abundant repeat in *Leishmania* genomes, TGC-microsatellite [41], was absent in *L*. *orientalis*, as also reported in the species of the subgenus *Viannia* [29]. Many other microsatellites such as C, GC, GTG, GGC, and GCG that might be categorized as uncommon for *Leishmania* [29] were noted in all samples of *Leishmania* species studied. In this study, size variants of the 3′-UTR fragments were partially due to a consequence of variations in the repeat number of the microsatellite sequences, the longer UTRs having a greater number of microsatellites. Although not seen within the isolate sequences examined in this study, such variations could also contribute to infraspecific variations in PCR product sizes.

Regarding species found outside Thailand, the *HSP70-I*-3′-UTR PCR generated products for *L*. *martiniquensis* and *L*. *orientalis* that were different in size from other tropical *Leishmania* as confirmed by sequencing. However, by gel electrophoresis alone the sizes of these PCR products could not reliably identify them, in particular *L*. *orientalis*. Therefore, PCR-RFLP analyses were carried out to attempt an improved level of discrimination. PCR-RFLP methods have been reported to be sometimes useful in providing improved *Leishmania* species discrimination compared to PCR alone, for example, using *Hae*III restriction fragments of a conserved region of the *Leishmania* minicircle kinetoplast DNA to distinguish *L*. *braziliensis* from *L*. *amazonensis* species [42]. Similarly, PCR-RFLP assays of ITS1 genes were used for direct identification of *L*. *major* and *L*. *tropica* [43]. In the current study the results of PCR-RFLP analyses in general corresponded to the prediction by *in silico* analyses of *Bsu*RI restriction fragments of the *HSP70-I*-3′-UTR amplified region. The PCR-RFLP patterns could clearly distinguish *L*. *martiniquensis*, *L*. *orientalis*, *L*. *braziliensis*, *L*. *amazonensis* and *L*. *major* species. Although different to the other species, the PCR-RFLP still could not distinguish two pairs of closely related species: *L*. *guyanensis* and *L*. *panamensis*; and *L*. *donovani* and *L*. *infantum*. However, previous studies have reported that PCR-RFLP patterns of the *HSP70* gene fragments digested with *Bcc*I restriction enzyme can differentiate between *L*. *guyanensis* and *L*. *panamensis* if required [44,45]. In this study, an unexpected band of >500 bp in both *L*. *guyanensis* and *L*. *panamensis* PCR-RFLP patterns was noted. The origin of this band is unclear but may have resulted from sequence variation in the *HSP70-I*-3′-UTR from these species. For *L*. *mexicana*, its pattern was similar to *L*. *aethiopica* and *L*. *tropica* but different from other species in the subgenus *Viannia* and *Leishmania*, including *L*. *amazonensis*, which is otherwise generally considered closely related to *L*. *mexicana*. However, in this case the results are due to co-incidence rather than any underlying relationship between *L*. *mexicana* and *L*. *aethiopica* or *L*. *tropica*. Overall, the combination of the PCR and PCR-RFLP analysis enabled clear identification of the two species that cause leishmaniasis in Thailand and discrimination of these from species found elsewhere in the world.

In Thailand, Sriworarat et al. (2015) have demonstrated a colorimetric loop-mediated isothermal amplification (LAMP) technique for the direct detection of *Leishmania* DNA. The LAMP assay could also detect DNA from multiple *Leishmania* species other than ‘*L*. *siamensis* (MHOM/TH/2010/TR)’ and *L*. *martiniquensis*, including *L*. *aethiopica*, *L*. *braziliensis*, *L*. *donovani and L*. *tropica* [46]. However, LAMP is prone to contamination due to the large amount of DNA that it can generate, and its capability to amplify minute amounts of DNA [46]. One PCR-based method using a pair of primers for minicircle kinetoplast DNA gene [27] can be used to discriminate *L*. *martiniquensis* from ‘*L*. *siamensis*’ (MHOM/TH/2010/TR) but the size of PCR products could not differentiate parasites in other subgenera [28]. For the ITS1-PCR method, it is not suitable for the discrimination of ‘*L*. *siamensis*’ (syn *L*. *orientalis*) and *L*. *martiniquensis* infection as it generates the same size of products [1,28,47].

The results of the detection limits in this study showed that the ITS1-rRNA PCR was the most sensitive method which could detect DNA of *L*. *martiniquensis* and *L*. *orientalis* at a concentration of 0.01 pg/μL compared to the *HSP70-I*-3′-UTR PCR and SSU-rRNA PCR methods which could detect DNAs of both species at the same DNA concentration (1 pg/μL). This can be explained in that the *HSP7*0 genes in *Leishmania* species studied so far are all arranged in a single genomic cluster that contains five or six *HSP70-I* copies followed by one *HSP70-II* copy [48], whereas copies of the SSU rRNA gene and the ITS1 region of the rRNA gene have been observed at between 20–40 copies per cell in *Leishmania* species [31,49].

The PCR-based methods used in this study can now be applied to the identification of *Leishmania* species obtained from vectors and reservoirs in Thailand to investigate their epidemiological significance. The technique is simple to perform and can be implemented in all settings where PCR-RFLP is available. For definite autochthonous cases, the PCR product alone may be sufficient for identification. However, where an imported case is suspected or cannot be eliminated, the *L*. *orientalis* PCR product is quite similar in size to that of *L*. *panamensis* and *L*. *guyanensis*, in which case the *Bsu*RI-PCR-RFLP method can be used for differentiating these species and some other species in other subgenera. However, where identification of species is essential or the infection is likely to have been acquired outside Thailand, sequencing of the *HSP70-I*-3′-UTR product or a similar discriminating target sequence is recommended. In conclusion, our data show that the 3′-UTR of *HSP70-I* region is a suitable target for PCR-based identification of and discrimination between *L*. *martinquensis* and *L*. *orientalis*.

## Supporting information

S1 Fig Agarose gel electrophoresis of *HSP70-I*-3′-UTR PCR products of *L*. *martiniquensis* isolates from Thailand and reference strain LEM2494. M = Molecular markers and N = Negative control.
(TIF)

S2 Fig Multiple sequence alignment of *HSP70-I*-3′-UTR sequences of *L*. *martiniquensis* isolates from Thailand and reference strain LEM2494. (TIF)

S3 Fig Multiple sequence alignment of *HSP70-I*-3′-UTR sequences of *L*. *amazonensis* (M2269, PH8 and C1S1) and *L*. *mexicana* (M379 and U1103) isolates. The positions of the *Bsu*RI (*Hae*III) restriction sites (GGCC) are highlighted.
(TIF)
