# Supplementary figures and images for: Molecular identification of two newly identified human pathogens causing leishmaniasis using PCR-based methods on the 3′ untranslated region of the heat shock protein 70 (type I) gene
Source: PLoS Negl Trop Dis. 2021 Nov 30;15(11):e0009982. doi: 10.1371/journal.pntd.0009982 (PMC8631652; doi:10.1371/journal.pntd.0009982)

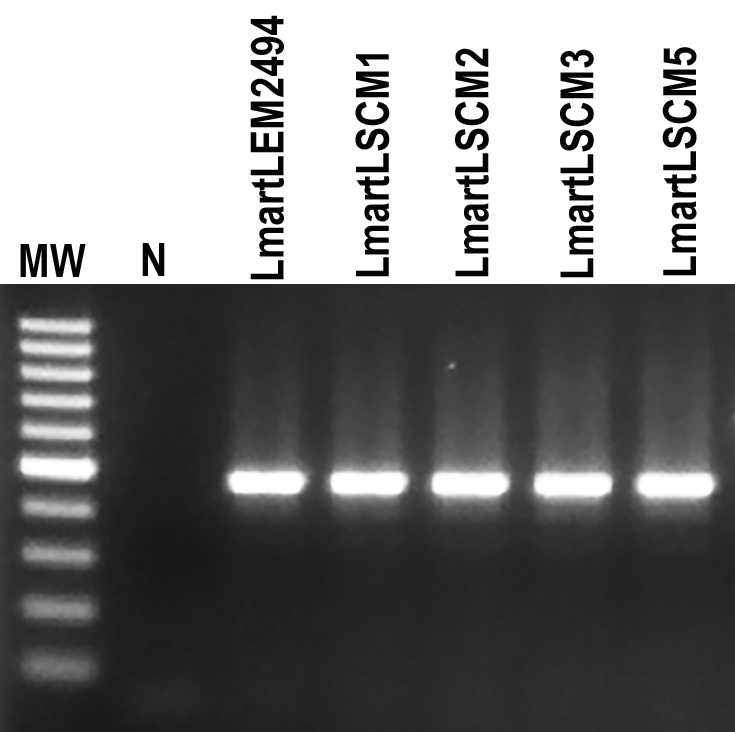

Supplement: S1 Fig — M = Molecular markers and N = Negative control. (TIF) [file pntd.0009982.s001.tif]

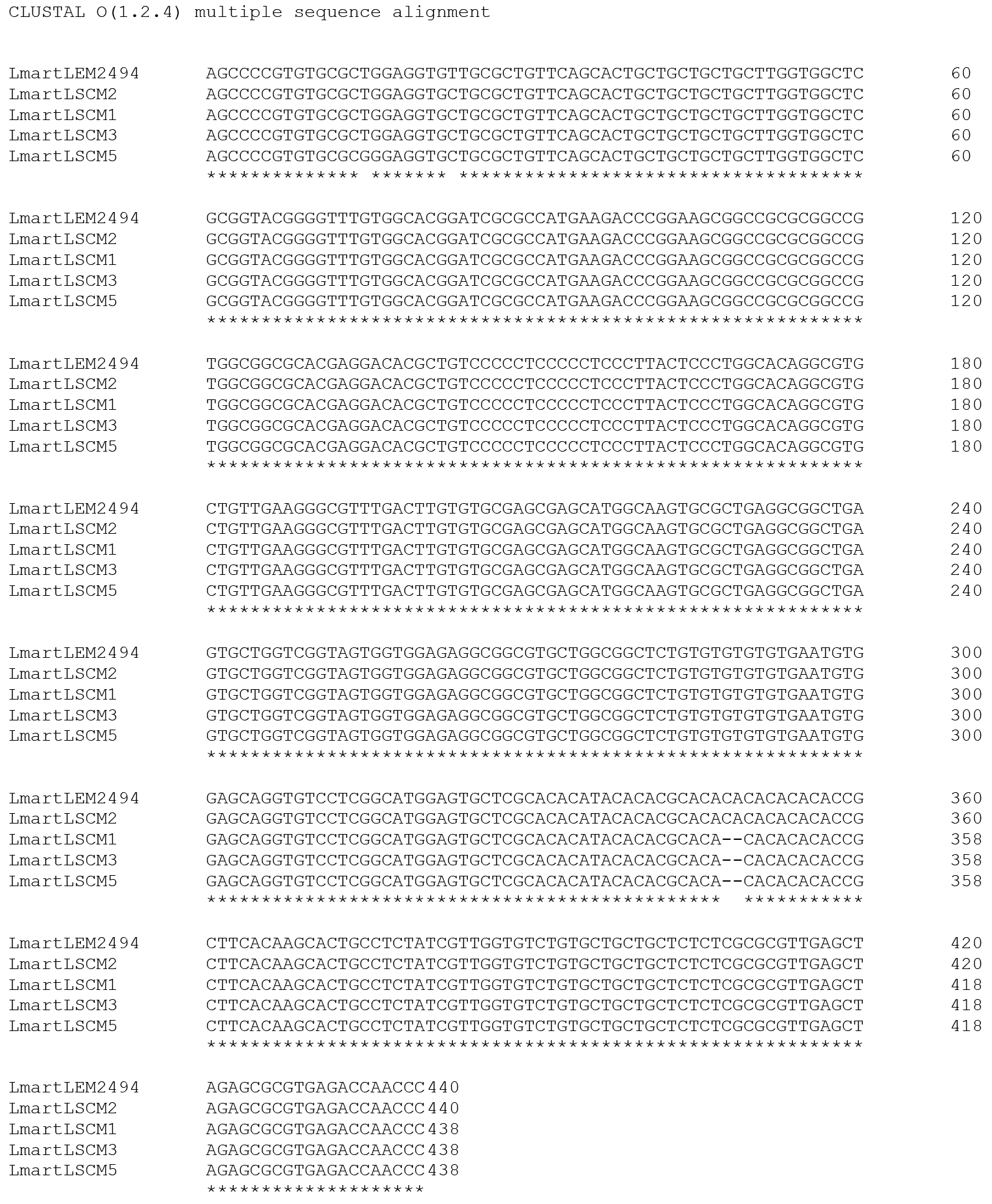

Supplement: S2 Fig — (TIF) [file pntd.0009982.s002.tif]

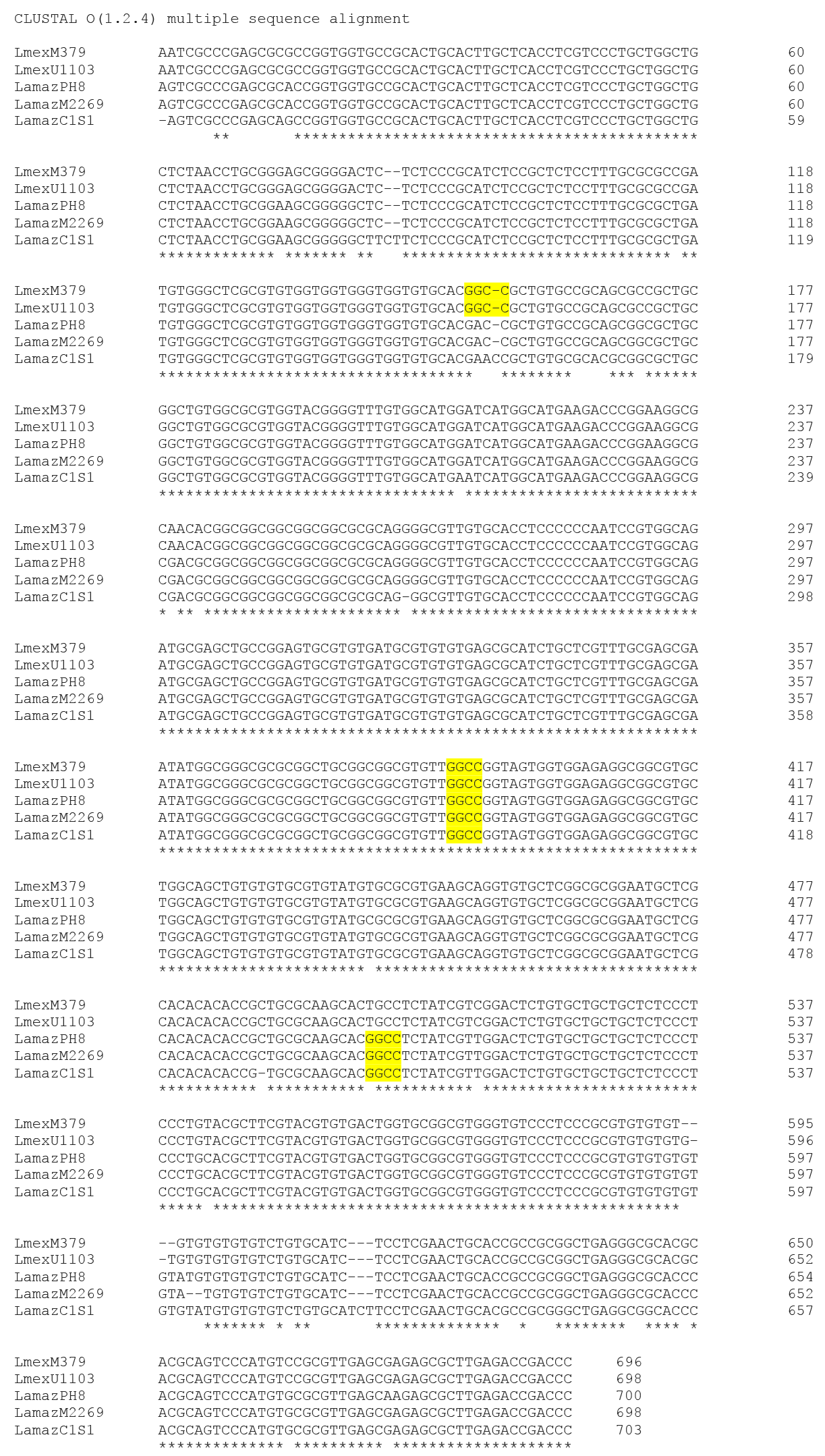

Supplement: S3 Fig — The positions of the BsuRI (HaeIII) restriction sites (GGCC) are highlighted. (TIF) [file pntd.0009982.s003.tif]
